# Macrophage reprogramming for therapy

**DOI:** 10.1111/imm.13300

**Published:** 2021-01-25

**Authors:** Valentina M. T. Bart, Robert J. Pickering, Philip R. Taylor, Natacha Ipseiz

**Affiliations:** ^1^ Systems Immunity Research Institute Cardiff University Cardiff UK; ^2^ Immunology Network Adaptive Immunity Research Unit GlaxoSmithKline Stevenage UK; ^3^ Department of Medicine School of Clinical Medicine Addenbrooke’s Hospital University of Cambridge Cambridge UK; ^4^ UK Dementia Research Institute at Cardiff Cardiff University Cardiff UK

**Keywords:** macrophages, polarization, reprogramming

## Abstract

Dysfunction of the immune system underlies a plethora of human diseases, requiring the development of immunomodulatory therapeutic intervention. To date, most strategies employed have been focusing on the modification of T lymphocytes, and although remarkable improvement has been obtained, results often fall short of the intended outcome. Recent cutting‐edge technologies have highlighted macrophages as potential targets for disease control. Macrophages play central roles in development, homeostasis and host defence, and their dysfunction and dysregulation have been implicated in the onset and pathogenesis of multiple disorders including cancer, neurodegeneration, autoimmunity and metabolic diseases. Recent advancements have led to a greater understanding of macrophage origin, diversity and function, in both health and disease. Over the last few years, a variety of strategies targeting macrophages have been developed and these open new therapeutic opportunities. Here, we review the progress in macrophage reprogramming in various disorders and discuss the potential implications and challenges for macrophage‐targeted approaches in human disease.

## Graphical abstract

Macrophages play roles at the heart of normal tissue physiology and in many diseases and show impressive capacity to adapt to their environment. Strategies to reprogramme macrophages represent new treatment options to be explored and exploited.

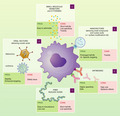

**Abbreviations**
AAV: adeno‐associated virus
AD: Alzheimer's disease
ALS: amyotrophic lateral sclerosis
BBB: blood–brain barrier
BMDM: bone marrow‐derived macrophage
CIA: collagen‐induced arthritis
CNS: central nervous system
DC: dendritic cell
EAE: experimental autoimmune encephalomyelitis
EGFR: epidermal growth factor receptor
FOXO3: forkhead box transcription factor family O 3
GA: glatiramer acetate
GM‐CSF: granulocyte–macrophage CSF
HDAC: histone deacetylase
ID3: inhibitor of DNA 3
IFN‐γ: interferon‐gamma
IL: interleukin
IRF5: interferon regulatory factor 5
KC: Kupffer cell
LXR‐α: liver X receptor‐alpha
mAB: monoclonal antibody
MBC: metastatic breast cancer
MCRPC: metastatic castration‐resistant prostate cancer
M‐CSF: macrophage colony‐stimulating factor
M‐CSFR: M‐CSF receptor
MHCI: major histocompatibility class I
MI: myocardial infarction
mo‐KC: monocyte‐derived KC
MS: multiple sclerosis
MФ: macrophage
NAFLD: non‐alcoholic fatty liver disease
NASH: non‐alcoholic steatohepatitis
NO: nitric oxide
NRF2: nuclear factor erythroid 2‐related factor 2
PDAC: pancreatic ductal adenocarcinoma
PGM: proneural glioblastoma multiforme
RA: rheumatoid arthritis
scRNAseq: single‐cell RNA sequencing
TAM: tumour‐associated macrophages
TGF‐β1: transforming growth factor beta 1
TLR: Toll‐like receptor
TNBC: triple negative breast cancer
TREM2: triggering receptor expressed on myeloid cells‐2
TTP: tristetraprolin
VEGF: vascular endothelial growth factor
Ywhaz: tyrosine 3‐monooxygenase/tryptophan 5‐monooxygenase activation protein zeta

## Introduction

Historically considered as broad‐range phagocytes playing a relatively ‘passive’ role within the immune system, macrophages (MФ) have since benefited from more recent intensive characterization. Present in almost every tissue, MФ have been divided into two broad subclasses: those derived from an embryonic progenitor and those from adult monocytes. Many tissue‐resident MФ have a prenatal origin (yolk sac‐ or fetal liver‐derived), their development is dependent on at least one essential tissue‐specific transcription factor, and they maintain themselves by self‐renewal, while others are recruited from the peripheral monocyte pool.^1^ The origins and replenishment of MФ populations have been the subject of numerous reviews^2^, ^3^, ^4^, ^5^, ^6^ and are not the focus of the present one. Tissue‐resident MФ are the predominant type of MФ present during steady state and are thought to monitor tissues, and maintain homeostasis, cellular communication and immune surveillance.^6^, ^7^ They also participate in developmental processes during embryogenesis.^8^, ^9^, ^10^, ^11^ Upon inflammation, whether induced by infection or injury, MФ are recruited in large numbers from circulating monocytes to the tissue and are often loosely classified as pro‐ or anti‐inflammatory. Previously, based on the actions of interferon‐gamma (IFN‐γ) and interleukin (IL)‐4 on MФ activation and with analogy to the Th1/2 T‐cell subsets, MФ were divided into two subtypes: ‘M1’ MФ, ‘classically activated’ by IFN‐γ; and ‘M2’ MФ, ‘alternatively activated’ by type 2 anti‐inflammatory cytokines such as IL‐10. Those two subtypes of MФ exhibit distinct metabolic function, the ‘M1’ having an anaerobic profile, based on glycolysis and production of nitric oxide (NO), whereas the ‘M2’ MФ have an aerobic one, based on oxidative phosphorylation and production of arginase.^12^, ^13^, ^14^ However, with current knowledge on MФ origin, diversity and significant plasticity,^15^, ^16^ the acceptance of this dogma has diminished. Recent cutting‐edge technological developments such as single‐cell RNA sequencing (scRNAseq), advanced animal genetic modification and intravital microscopy^17^, ^18^, ^19^, ^20^ have allowed for a greater appreciation of cellular diversity than previously acknowledged. MФ not only have impressive variability in their gene expression but they are also phenotypically plastic, allowing them to adapt to their environment and ensure appropriate responses. Tissue‐specific transcriptional programmes, instigated by local signals, enable phenotypic specialization in discrete microenvironmental niches controlled by differential transcription factor usage.^21^, ^22^ Dysfunction of MФ behaviour or phenotype has been associated with the development of many conditions such as neurodegeneration, arthritis, chronic inflammation, atherosclerosis and cancer,^23^, ^24^ and the identification of distinct subpopulations of MФ may be key in disease understanding and treatment.^25^, ^26^, ^27^

All MФ rely on specific cytokine availability for survival, proliferation and phenotype, including macrophage colony‐stimulating factor (M‐CSF), granulocyte–macrophage CSF (GM‐CSF), IL‐34 and transforming growth factor beta 1 (TGF‐β1), as single factor or in combination.^6^, ^28^, ^29^, ^30^ MФ programming strongly depends on the environment, which profoundly affects the cells at a transcriptomic and epigenetic level.^21^, ^22^, ^31^ MФ also demonstrate remarkable immune memory or ‘trained immunity’ capacities^32^, ^33^, ^34^, ^35^ based on epigenetic modification following stimulation and long‐term priming or ‘imprinting’, as seen with apoptotic cell recognition leading to a relatively stable tolerant state.^36^, ^37^, ^38^ This immune memory appears critical during transplant, when monocytes and MФ acquire specific memory to major histocompatibility class I (MHCI).^39^ However, once isolated for *ex vivo* study, the characteristic gene signature of tissue‐resident MФ, including epigenetic modification,^22^, ^40^, ^41^ is often lost, indicating the need to explore their function and reprogramme them preferentially *in vivo*, or with the *in vivo* context considered.

The aim of this review was to have a broad overview of current research and possible treatments directed at MФ in a selection of disease contexts. We have confined the focus to specific organs and conditions detailed below, purely as exemplars of the kinds of approaches that are being considered. However, the potential for MФ targeting should not be considered restricted to the provided examples.

## Methods to reprogramme macrophages

**Figure 1:**
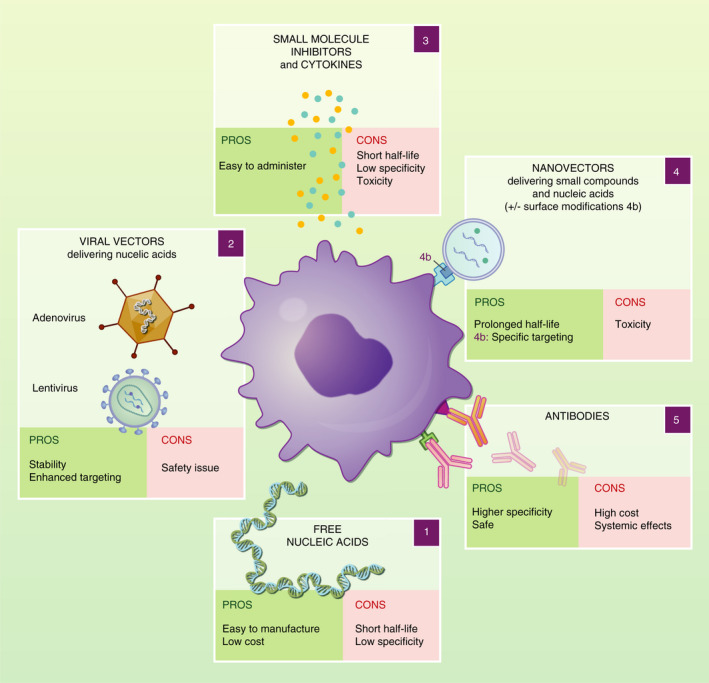
Summary of macrophage manipulation techniques for therapeutic purpose. These strategies can directly be applied *in vivo*, as well as *in vitro* followed by adaptive transfer of manipulated MФ. Free nucleic acids (1) can be manufactured easily and are very successfully used in some tissues including lungs and skeletal muscle. However, they lack MФ specificity and are rapidly cleared from the environment, mostly by circulating enzymes and kidney. Viral vectors (2) can be employed to deliver nucleic acids, preventing clearance from the system. Depending on the type of vector, gene manipulation can be long term (lentivirus) or transient (adenovirus). Viral vectors are highly efficient and can be modified to improve MФ targeting. However, they do entail safety considerations for patients and manufacturing staff. Free small molecules and cytokines (3) are known to act on MФ polarization. They are easy to administer but prone to degradation. They are also often not MФ specific and can cause off‐target effects and toxicity. Encapsulation of nucleic acids, small molecules and cytokines into nanovectors (4) prolongs their half‐life in the organism, while surface modifications (4b) allow targeting of specific cell types. Antibodies (5) can manipulate MФ polarization by directly binding Fc or other cell surface receptors. While they are generally safe, high doses are often required for therapeutic efficacy translating into high costs

MФ exhibit a high degree of plasticity in response to environmental signals, many of which are tissue‐ and context‐specific. This results in a variety of MФ subtypes with different origins, which may play distinct roles in human disease and potentially provide unique opportunities for targeted therapies.^26^, ^27^, ^42^ Before considering detailed examples of therapeutic approaches in specific tissue and disease contexts, we briefly introduce some common methods used to reprogramme MФ, from conventional approaches, such as targeted antibody treatments and small molecule drugs to cutting‐edge technology of gene expression modification using viral vectors, artificial DNA carriers, naked DNA and cell therapy (Figure 1).

Targeted antibody treatments are among the easiest and most efficient methods to target not only MФ surface receptors involved in the regulation of immune responses^43^ but also circulating cytokines/growth factors, preventing their interaction.^44^ As a result, antibodies can alter MФ activation status. However, this technique is mostly systemic and can lead to numerous off‐target effects.

Gene therapy aims to alter specific gene expression by inserting genetic material into the target cell. Free nucleic acids can be directly injected *in vivo*. While this is generally considered safe,^45^ detection by MФ can induce inflammatory signalling. Although possibly advantageous when reprogramming MФ into pro‐inflammatory phenotypes, it may counter anti‐inflammatory states and raises concern of off‐target effects. Free nucleic acids also lack cell‐targeting specificity, an issue that can be solved by attachment to carrier molecules, such as coupling to peptides directly targeting MФ cell surface receptors.^46^

Nucleic acids can also be introduced using modified viral vectors that lack the genes necessary for replication. Lentiviral vectors stably integrate genetic material into the host cell genome, while adenoviruses and adeno‐associated viruses (AAVs) only cause transient gene expression. Despite their high efficacy, viral vectors are associated with significant disadvantages: from a manufacturing perspective, viral vector production is costly and requires specific safety measurements.^47^ From a clinical point of view, random lentiviral RNA insertion into the genome could cause tumour suppressor gene disruption triggering malignancy.^48^ Also, viral vectors bear the risk of potentially high immunogenicity.^49^, ^50^ Adenoviruses’ triggering of immune responses could, however, be exploited for the use in tumour contexts where immune activation could be beneficial.

Another option for delivery of nucleic acids are non‐immunogenic nanoparticles (<100 nm), and organic (e.g. liposome, polymers) or inorganic (e.g. gold, silica) particles widely used in clinical applications^51^ that are readily ingested by MФ. Different types of nanovectors vary in their advantages in clinical applications^52^ and can inherently favour polarization towards either end of the MФ activation spectrum.^53^

The use of nanoparticles in not restricted to nucleic acid delivery. MФ can also be targeted with compounds acting on signalling pathways to promote polarization such as cyclooxygenase‐2 inhibitors,^54^ receptor tyrosine kinase inhibitors^55^ and histone deacetylase (HDAC) inhibitors,^56^ as well as small molecule Toll‐like receptor (TLR) agonists^57^ and possibly cytokines.^58^ Free small molecules can be associated with systemic side effects, while unprotected molecules and cytokines are relatively unstable *in vivo*.^59^ Such limitations can be overcome by encapsulation into nanovectors.^60^, ^61^ In general, nanoparticles are well tolerated, small enough to cross physiological barriers including the blood–brain barrier (BBB) and easily modified to allow cell‐targeted delivery. They do, however, need to be carefully manufactured considering possible toxicity associated with different materials and delivery routes, as well as inflammatory responses associated with uptake by MФ.^62^

Blood and bone marrow‐derived MФ (BMDM) can be reprogrammed *ex vivo* by the same aforementioned methods and adoptively transferred to individuals. This may help alleviate some of the off‐target limitations of directly targeting MФ *in vivo*.^63^ To retain polarization stability, MФ can be genetically engineered *ex vivo* to over‐ or under‐express factors associated with polarization phenotypes. An intriguing option was proposed by Shields and colleagues: *ex vivo* attachment of IFN‐γ‐loaded phagocytosis‐resistant ‘backpacks’ to BMDM enabled slow release of IFN‐γ *in vivo*, allowing injected cells to maintain a pro‐inflammatory phenotype while simultaneously polarizing tumour‐infiltrating MФ.^64^

The type of protocol used for therapeutic intervention will depend on the tissue, MФ subtype and pathology to be treated. Recently, a first‐in‐human phase 1 dose–escalation trial confirmed the safety of autologous MФ therapy in end‐stage liver disease, which was well tolerated and is currently undergoing efficacy measures in an ongoing phase 2 randomized controlled trial.^65^

## Tissue‐specific consideration in macrophage reprogramming

### Cardiac macrophages

Mouse cardiac MФ are composed of four different subsets, distinguishable by their cell surface expression of typical markers such as MHCII, Ly6C, CCR2, CD11c, MerTK, CD206 and CD64, and are derived from yolk sac and fetal liver.^66^ Their self‐renewal capacity decreases with age, and they are thought to be gradually replenished by monocyte‐derived MФ over time.^67^ Cardiac MФ play important roles in tissue homeostasis, angiogenesis and vascular remodelling during embryogenesis, as well as in the action potential propagation by being electronically coupled with cardiomyocytes.^68^, ^69^, ^70^ During inflammation or following injury such as myocardial infarction (MI), resident cardiac MФ become activated and high numbers of monocyte‐derived MФ are recruited to the injured site where their heterogeneity is thought to impact MI outcome.^71^, ^72^, ^73^ Recruited cardiac MФ participate in all phases of MI, from the first acute inflammatory wave to the reparative phase and the promotion of angiogenesis and muscle regeneration.^74^, ^75^ The repair after MI is associated with scar formation and fibrosis, decreasing cardiac functionality.^76^ Interestingly, the resident cardiac MФ seem to limit adverse remodelling^77^ and Gata6^+^ pericardial MФ have recently been shown to enter the site of injury and prevent fibrosis.^78^ MI is a leading cause of death worldwide.^79^ Clinical trials mainly focus on decreasing systemic inflammation by suppressing the immune system and inflammation with corticosteroid,^80^ methotrexate (phase 3, completed and recruiting) or blocking pro‐inflammatory molecules such as IL‐6 (tocilizumab, phase 2, active) and IL‐1β (Anakinra, completed^81^). However, the use of broad‐range immunosuppressors is questionable, as a controlled first phase of inflammation has been shown to be essential to tissue regeneration.^82^ In this line, recent work has shown that a located injury such as MI not only modifies the local MФ pool, but also affects the number and molecular signature of off‐site MФ throughout the organism (liver, lung and kidney),^83^ placing the effect of systemic drugs in an even more central question. Recent research focusing on the modulation of cardiac MФ phenotype and activity in animal models shows promising results encouraging further human clinical trials. Using nanoparticle‐delivered siRNA, Courties et al. successfully silenced interferon regulatory factor 5 (IRF5) in cardiac MФ in a mouse model of MI, which improved healing.^84^ Similarly, the targeting of miRNA‐21 in cardiac MФ with nanoparticles containing mimics (intravenously injected) or adenovirus particles overexpressing miR‐21 (intramyocardially injected) reduced inflammation, fibrosis and cardiac dysfunction.^85^, ^86^ Finally, the intramyocardial transplantation of *in vitro* M‐CSF‐ and IL‐4‐primed ‘reparative’ MФ following MI in mice showed beneficial effects over transplantation of non‐primed bone marrow mononuclear cells.^87^ While human assays to modify MФ *in vivo* has been less prominent, recent work explored the MФ heterogeneity and identified at least two subsets (CCR2^+^ and CCR2^‐^) present in human heart which are essential for tissue function.^88^

### Macrophages in the central nervous system

Resident myeloid cells in the central nervous system (CNS) include parenchymal microglia and different types of non‐parenchymal MФ.^89^ Additionally, inflammatory conditions can trigger the influx of monocyte‐derived MФ^90^ through disruption of the BBB. Under physiological conditions, this layer of endothelial cells restricts the crossing of cells and compounds into the CNS as previously reviewed.^91^ Both dysregulated resident and infiltrating MФ have been linked to the pathophysiology of several neurological disorders including Alzheimer's disease (AD),^92^, ^93^ multiple sclerosis (MS)^94^ and amyotrophic lateral sclerosis (ALS).^95^ Currently, no cure exists for those conditions and potential preventative and early treatment options face the difficulty of drug delivery.

#### Multiple sclerosis

MS is an autoimmune, demyelinating disease of the brain and spinal cord. It affects young adults causing progressive neurological deterioration with common symptoms including numbness, burning sensations, visual impairment, loss of balance, bladder dysfunction, fatigue and depression.^96^ While the aetiology of MS is incompletely understood, it is believed that CNS‐infiltrating pro‐inflammatory phagocytes are key drivers in tissue destruction.^94^ Indeed, in MS patients such cells have been found to express reduced levels of SHP‐1 causing increased activation of STAT1, STAT6 and NF‐κB.^97^ This was associated with a pro‐inflammatory phenotype characterized by elevated proteinases including ADAM8, which could disrupt the BBB and contribute to demyelination, as well as increased molecules associated with antigen presentation and costimulation.^98^ This view is supported by studies of experimental autoimmune encephalomyelitis (EAE), the murine model for MS.^99^ Depletion of infiltrating phagocytes protected mice form axonal damage,^100^ while blocking microglial release of nitrite and pro‐inflammatory chemokines and cytokines significantly reduced clinical signs in the EAE model.^101^ The polarization of microglia by molecular control of cytokines and costimulatory molecules by nuclear receptor has been shown to be essential for the onset and development of EAE.^102^ EAE has limitations concerning translation to human disease but does still play an important role in drug development.^103^

Glatiramer acetate (GA) is a polymer approved for the treatment of relapsing–remitting MS. While its mode of action is attributed to T‐cell manipulation, GA also increases microglial phagocytic activity and IL‐10 production while decreasing TNF *in vitro*.^104^ GA likely does not cross the BBB on its own. However, it can be taken up by dendritic cells (DCs) and released in the CNS, with GA uptake promoting trans‐endothelial migration of DCs.^105^ Alternatively, GA could enter the CNS when the BBB is disrupted in active MS lesions. Studies report GA‐mediated reductions in relapse rates;^106^ however, it is still unclear whether its effect on microglia contributes to this and whether GA actually slows disease progression.^107^ As GM‐CSF has been implicated in disease induction in the EAE model, MOR103, a monoclonal antibody (mAb) directed against GM‐CSF, has been tested in MS patients and was found to be well tolerated, although no assessment regarding efficacy was conducted at this stage (NCT01517282).

While infiltrating pro‐inflammatory phagocytes are potentially detrimental in MS, microglial production of pro‐inflammatory factors, especially TNF, might support remyelination,^108^ calling for a specific targeting of peripheral MФ. Indeed, preventing peripheral MФ entry into the CNS was beneficial in EAE.^109^

Therapeutics aiming to interfere with peripheral MФ need to be carefully considered to not affect microglia populations. In EAE, scRNAseq recently identified eight different monocyte subsets, one of which expressing CXCL10^+^ was associated with a pathogenic gene signature. This subset had also been observed in other inflammatory models such as pathogen infection, and its depletion in EAE was associated with clinical improvement.^110^ Whether and how this finding translates into the human context remains to be investigated.

#### Alzheimer's disease

AD is a chronic neurodegenerative disorder associated with CNS inflammation and the main cause of dementia. Genome‐wide association studies have uncovered a variety of loci increasing susceptibility to the development of late‐onset Alzheimer’s disease, many of which are associated with immunity.^93^ In addition, rare coding variants, such as that of triggering receptor expressed on myeloid cells 2 (TREM2), have strongly been linked to increased risk of developing AD.^111^ In line with this, in a murine AD model expressing a TREM2 risk variant (R47H), anti‐human TREM2 mAb increased microglial proliferation, reduced neuroinflammation and was associated with cognitive benefits.^112^ The same antibody was well tolerated in a phase I clinical trial (NCT03635047).

Activated microglia are thought protective early in disease but over time become dysfunctional causing inflammatory injury.^113^ Importantly, scRNAseq identified distinct microglia subsets in AD, including a type associated with neuroprotection and increased phagocytosis also found in a mouse model of ALS.^25^

An important regulator in microglial differentiation is the M‐CSF receptor (M‐CSFR): depending on the murine model and dose of inhibitor used, inhibition of M‐CSFR signalling resulted in blockade of microglial proliferation^114^, ^115^ or microglial depletion^116^ inducing an anti‐inflammatory state and cognitive improvements not associated with alterations in amyloid plaques. Together, these and other studies suggest M‐CSFR inhibition as a viable option for clinical trials. Importantly, M‐CSFR expression is not restricted to microglia but important for all macrophages. In line with this, M‐CSFR inhibition was shown to also affect circulating and tissue‐resident macrophages and lymphocytes in other organs in a mouse model.^117^ In this paper, the M‐CSFR inhibitor PLX5622 was administered through diet. While not investigated by the authors, it is likely that such a method of drug delivery would also affect the gut microbiome. Considering the link between intestinal dysbiosis and neurodegeneration,^118^ such a method could affect treatment outcomes and a more localized way of drug delivery, potentially by intranasal^119^ or direct intraventricular/intrathecal administration,^120^ could minimize off‐target effects.

#### Amyotrophic lateral sclerosis

ALS is characterized by motoneuron degeneration typically causing paralysis and death within five years of diagnosis. In murine models, the number of resident microglia increases throughout disease progression concomitant with a switch from an anti‐ to a pro‐inflammatory phenotype.^121^ Simultaneously, peripheral monocytes from ALS patients have been suggested to be more readily activated into pro‐inflammatory phenotypes compared with those of healthy individuals, further driving neuroinflammation after CNS infiltration.^122^

Vascular endothelial growth factor (VEGF) has been suggested to play a neuroprotective role by reducing motor neuron death through downregulation of pro‐inflammatory cytokine production.^123^ Intrathecal injection of adeno‐associated virus containing VEGF expressing plasmid induced VEGF expression in motor neurons in a mouse model of ALS. This prolonged survival of the mice by two modes of action: increasing anti‐apoptotic factors such as Bcl‐2 and decreasing pro‐apoptotic ones such as Bax, caspase‐3 and caspase‐9 in neurons and a switch in the inflammation balance (reduction in the pro‐inflammatory TNF, IL‐1β and CD68 while increase in anti‐inflammatory TGF‐β released by microglia).^124^ Unfortunately, phase II clinical trials for intracerebroventricular administration of the common splicing isoform VEGF165 were terminated due to a lack of favourable benefit–risk ratio (NCT01384162).

The involvement of pro‐inflammatory MФ and microglia in the pathophysiology of neuroinflammatory conditions strongly suggests the targeting of CNS myeloid cells for re‐education as a treatment option. However, to date no agent designed to manipulate MФ activation states in the CNS has been approved for the treatment of a neurological condition. This is partly due to poor animal models, which do not fully mimic human pathophysiology. It is essential to understand the heterogeneity of myeloid populations and individual contributions to neurological pathologies in the CNS, to specifically target disease‐associated cell types. Imaging mass cytometry is already used to characterize key players in neurological diseases by applying antibodies to post‐mortem sample.^125^ Additionally, scRNAseq of human microglia from brain autopsy samples could also uncover cell‐specific targets for intervention that could aid the development of therapeutics. With potential targets being discovered, it is vital to also reconsider options for drug delivery: compounds delivered systemically can eventually reach the brain but will also affect cells in other tissues and organs. In particular, in the case of macrophage manipulation, many receptors and targets are shared by different tissue‐resident macrophage populations making it difficult to prevent off‐target effects. This issue could be overcome by exploring intracerebral,^126^ interstitial^127^ or intranasal^128^ delivery methods.

### Liver macrophages

The murine liver comprises two distinct populations of tissue‐resident MФ, the Kupffer cells (KCs) that occupy the sinusoidal vascular space, and the phenotypically distinct liver capsular MФ, which reside in the hepatic capsule.^129^ Additionally, the liver may also contain monocyte‐derived and peritoneal MФ,^86^ which are recruited following inflammatory events or injury.^130^, ^131^, ^132^ Liver MФ heterogeneity was recently reviewed.^130^, ^131^ In mice, fetal liver monocytic precursors give rise to embryonic KCs, which at steady‐state maintain the KC pool through self‐renewal, independent of BM‐derived progenitors.^1^, ^133^, ^134^ In contrast, liver capsular MФ arise entirely from adult circulating monocytes.^129^ More recently however, BM‐derived monocytes were shown to populate the KC niche during postnatal liver development, contributing significantly to the adult KC population.^135^ Following the loss of KCs after infection,^136^ or experimental depletion,^135^, ^137^, ^138^ the KC pool is repopulated through proliferation of surviving KCs, as well as the recruitment and differentiation of blood monocytes into KCs (mo‐KCs). These mo‐KCs were shown to be highly functionally and transcriptionally homologous to their embryonic counterparts^135^ after 30 days post‐depletion, although other studies have shown that embryonically derived KCs may exhibit some phenotypic and functional differences to their monocyte‐derived counterparts.^137^, ^139^ The replenishment of the depleted KC niche by either mechanism however, appears to be context‐dependent. Indeed, in an acute liver injury model, the depleted KC niche was replenished through proliferation of the surviving KCs without input from circulating monocytes.^140^ Similar to the development and maintenance of other tissue‐resident MФ populations, recent studies have highlighted the critical role of transcription factors in governing KC development and identity, with the loss of inhibitor of DNA 3 (ID3)^141^ or liver X receptor‐alpha (LXR‐α)^142^ resulting in KC deficiency in mice. Two recent studies in mice demonstrated that liver‐derived signals orchestrate monocyte recruitment^143^ and initiate and maintain KC identity through the induction of lineage‐determining factors including ID3 and LXR‐α by acting on pre‐existing but poised enhancers^143^, ^144^. Additionally, leveraging of scRNAseq technology for profiling human liver MФ has revealed distinct subpopulations of KCs with discrete gene expression signatures^26^, ^145^, ^146^, ^147^, but our understanding of their biology is still restricted compared with mouse liver MФ.

Liver MФ play a key role in the pathogenesis of acute and chronic liver diseases, including acute liver failure, alcoholic liver disease, non‐alcoholic fatty liver disease (NAFLD), viral hepatitis and hepatocellular carcinoma. They are also critically required for the restoration of tissue homeostasis and resolution of liver disease.^148^ These seemingly contrasting roles for liver MФ highlight the importance to identify and understand the relative contributions that distinct subsets have in disease progression, as well as tissue repair, to help the development of improved‐targeted therapeutics, as well as the definition of biomarkers indicating either disease progression or regression (for a recent review, see Ref 131).

A recent study described a subset of Trem2^hi^ MФ enriched in mouse models of non‐alcoholic steatohepatitis (NASH), as well as in human NASH livers, correlating with disease severity.^149^ Interestingly, in a diet‐induced mouse model of NASH, KC identity was significantly altered by the NASH diet. NASH‐induced changes in KC enhancers and gene expression were driven by activator protein 1 and early growth response protein 1 inducing a scar‐associated MФ phenotype, with increased expression of both *Trem2* and *Cd9*.^150^ Interestingly, an independent study also identified a scar‐associated TREM2^+^CD9^+^ subpopulation of MФ in humans, which differentiate from circulating monocytes and expand during liver fibrosis.^26^ In another study, a subset of MФ (MerTK^+^HLA‐DR^high^) was reported to expand during the resolution phase of acute liver disease, with a comparable population identified in mice during the resolution phase of an acute liver injury model.^151^ Further studies are needed to fully define liver MФ subsets and their contributions to disease progression, as well as in tissue repair and the restoration of homeostasis. However, some approaches to target MФ in liver disorders are already under investigation.

#### Glucocorticoid and antibody–drug conjugate

Glucocorticoid receptor signalling modulates inflammation in KCs, suggesting that glucocorticoid treatment could serve as a potential MФ‐directed treatment for liver diseases.^152^ Liposomal delivery of dexamethasone was shown to significantly reduce liver injury and fibrosis in experimental models of both acute and chronic liver injuries.^153^ Direct targeting of MФ with an antibody–drug conjugate composed of dexamethasone linked to an antibody against CD163 (a scavenger receptor highly expressed in KCs and infiltrating monocytes/MФ) was shown to reduce inflammation, hepatocyte ballooning and fibrosis in a mouse model of NASH, while having no apparent systemic side effects.^154^

#### Cytokines modulation

The pro‐inflammatory cytokine TNF plays a major part in the development of steatosis, inflammation and fibrosis in NAFLD. KCs have been identified as the main cellular source of TNF in mouse models of NAFLD. Mannose‐modified trimethyl chitosan–cysteine‐conjugated nanoparticles were used to deliver siRNA targeting TNF to MФ and protected mice from inflammation‐driven liver damage and lethality in an acute liver injury model.^155^

#### Gene expression modification

Oxidative stress and the associated damage have been suggested to link obesity and liver disease. Recently, Azzimato and colleagues^156^ showed that oxidative stress was triggered by obesity in mouse and human livers. In parallel, nuclear factor erythroid 2‐related factor 2 (NRF2), a transcription factor regulating the antioxidant response, was reduced, leading to an impaired antioxidant response. miR‐144 was greatly upregulated in the liver of obese and insulin‐resistant mice and humans and was shown to target NRF2. Consequently, delivery of an antagomiR targeting miR‐144 expression to MФ *in vivo* with glucan‐encapsulated RNA interference particle technology increased NRF2 protein levels, reduced oxidative stress and improved hepatic metabolism in insulin‐resistant mice.

#### Small molecule inhibitors

Galectin‐3 is a pleiotropic protein highly expressed by MФ in the liver and upregulated in models of liver disease.^157^, ^158^ Galectin‐3 deficiency in mice was protective in a concanavalin A‐induced liver injury model, reducing pro‐inflammatory cytokines, as well as attenuating fibrosis.^159^ Similarly, pharmacological inhibition of Galectin‐3 in liver injury models significantly reduced fibrosis and led to a reversal in cirrhosis and is now under evaluation for NASH in clinical trials.^157^, ^158^

The dual CCR2/CCR5 inhibitor cenicriviroc was shown to reduce monocyte recruitment and liver injury in an acute liver failure model,^160^ as well as ameliorating hepatic inflammation and fibrosis in experimental models of NASH,^161^ and is also currently under evaluation in clinical trials for the treatment of liver fibrosis in NASH (NCT03059446 and NCT03028740).^162^

#### Adoptive transfer

In an experimental model of acute liver injury, adoptive transfer of *ex vivo* IL‐4/IL‐13‐polarized BMDM rapidly reduced liver injury and several mediators of inflammation.^163^ Of note, the adoptive transfer of primary human‐polarized monocyte‐derived MФ partially recapitulated the therapeutic effect observed with polarized mouse BMDM in the same mouse model.^163^ Similarly, injection of BMDM or embryonic stem cell‐derived MФ reduced both fibrosis and improved liver regeneration in a hepatic injury and fibrosis model.^164^, ^165^ Clinical trials in humans have demonstrated the safety of administration of large and frequent infusions of autologous MФ. A recent first‐in‐human trial evaluated the safety of a single peripheral infusion of autologous MФ in end‐stage liver disease, which was well tolerated and led to a reduction in clinical scoring, and is currently undergoing efficacy measures in an ongoing phase 2 randomized controlled trial.^65^ However, this study did not determine whether the infused MФ migrated to and engrafted in the liver. Previous studies in mice, as well as a case study in humans, suggest that the administration via peripheral or central veins MФ traffic from the pulmonary vasculature via the blood before engrafting in the liver and spleen.^164^, ^166^, ^167^, ^168^ Significant challenges remain for the adoption of autologous MФ therapies, such as their scalability, as well ensuring that engrafted cells maintain the intended phenotype, which can be greatly impacted by the tissue microenvironment.

### Macrophages in arthritis

Rheumatoid arthritis (RA) is a chronic, systemic inflammatory autoimmune disorder that primarily affects synovial joints, leading to irreversible bone and cartilage destruction. Multiple studies have implicated monocytes and MФ in the initiation and progression of RA.^169^ MФ are the most abundant immune cell and are a source of pro‐inflammatory cytokines associated with RA pathogenesis including TNF, IL‐6 and IL‐1β,^170^ as well as chemo‐attractants and metalloproteinases. Conversely, synovial tissue MФ in healthy and RA patients in sustained remission suggest that MФ have a fundamental role in maintaining and/or reinstating synovial homeostasis.^171^, ^172^ In a murine model of sterile inflammatory arthritis, non‐classical Ly6C^‐^ but not Ly6C^+^ monocytes were reported to be crucial for the initiation of arthritis, while tissue‐resident synovial MФ restricted the development of arthritis.^173^ Murine synovial lining CX3CR1^+^ MФ form an immunological barrier in the lining layer of the synovium of healthy joints.^174^ Depleting them in a mouse model of arthritis disrupted barrier function and accelerated the onset and magnitude of arthritis, whereas depletion of CSF1R^+^ monocytes and MФ expedited the resolution of inflammation. Interestingly, comparison of scRNAseq data with human data sets from RA patients revealed significant overlap, suggesting cells similar to synovial lining MФ may also exist in humans.^174^ A recent study described synovial MФ subsets enriched in active RA (MerTK^‐^ CD206^‐^) or sustained remission (MerTK^+^ CD206^+^), which are thought to contribute to RA pathogenesis or remission through the production of pro‐inflammatory cytokines or lipid mediators, respectively.^175^ Similarly, *IL1B^+^* pro‐inflammatory monocytes were enriched in synovial tissue from patients with RA, whereas *NUPR1^+^* monocytes were inversely correlated with tissue inflammation.^176^ Another study recently identified HBEGF^+^ inflammatory MФ enriched in the human RA tissues,^177^ which promote synovial fibroblast invasiveness in an epidermal growth factor receptor (EGFR)‐dependent manner. Interestingly, a previous study showed that EGFR inhibition reduced the severity of established RA in mice.^178^ Collectively, these studies demonstrate that distinct monocyte and MФ subsets have defined roles in RA. Strategies are currently being developed to target MФ and improve RA pathology.

#### Cytokines

Several lines of evidence suggest that MФ and GM‐CSF strongly influence the development and progression of RA.^179^, ^180^ In patients with RA, GM‐CSF is elevated in plasma, synovial fluid and synovial tissues, while administration of recombinant GM‐CSF has been reported to exacerbated RA disease activity.^181^ Depletion of GM‐CSF or blockade of GM‐CSFRα in a mouse model of RA significantly reduced the number of MФ in the inflamed synovium, decreased synovial inflammation and joint destruction.^182^, ^183^ Therapeutic antibodies targeting GM‐CSF and its receptor have been developed and evaluated in clinical trials.^181^, ^184^ Collectively, these studies have shown that treatment is associated with rapid and sustained improvements in measures of RA disease outcomes, as well as being well tolerated in safety studies.^185^ Of note, a phase III clinical trial is currently ongoing evaluating otilimab, a fully human anti‐GM‐CSF monoclonal antibody, in RA patients who have had an inadequate response to disease‐modifying anti‐rheumatic drugs and/or Janus kinase inhibitors (NCT04134728).

#### Gene therapy

In the collagen‐induced arthritis (CIA) mouse model, Kong and colleagues identified three key pro‐resolving factors that were elevated in mouse synovial tissues during the resolution phase of CIA.^186^ Among them, tyrosine 3‐monooxygenase/tryptophan 5‐monooxygenase activation protein zeta (Ywhaz) was also shown to be elevated in rat synovial tissue in a CIA model during the resolution phase compared with the peak phase, as well as in RA patients who responded well to treatment with anti‐rheumatic drugs. *Ywhaz* is highly expressed in Tregs and MФ and is one of the most common proteins found in exosomes^187^ from Tregs, although no data were shown for MФ in this study. Interestingly, treatment of thioglycollate‐elicited peritoneal MФ with recombinant Ywhaz reduced the expression of *Tnf* and *Il6*, while enhancing *Il10* expression.^186^ Furthermore, siRNA knockdown of *Ywhaz* or treatment with an anti‐Ywhaz antibody enhanced the expression of *Tnf* and *Il6* following LPS stimulation, supporting a role of Ywhaz in modulating pro‐inflammatory cytokine production in MФ.^186^ Intra‐articular delivery of adenovirus expressing Ywhaz suppressed the production of pro‐inflammatory cytokines and significantly reduced synovial inflammation and joint destruction in mice.^186^ Ywhaz is a member of the 14‐3‐3 protein family, which modulate the activity of binding partners by controlling protein localization, stability, conformation and activity mainly through phosphoserine/threonine motifs.^188^ Previous studies have suggested that Ywhaz can modulate the activity of forkhead box transcription factor family O 3 (FOXO3), a transcription factor regulating the expression of cytokines,^189^ and tristetraprolin (TTP),^190^ which regulates cytokine production by destabilizing target mRNA molecules.^191^ However, further studies are needed to understand how Ywhaz regulates the production of cytokines and the subsequent resolution of arthritis. In arthritic rats, MФ phenotype has been manipulated *in vivo* by tuftsin‐modified nanoparticle‐mediated delivery of plasmid encoding IL‐10, leading to a significant reduction in pro‐inflammatory cytokine production, diminishing inflammation and preventing the progression of joint damage.^192^

### Tumour‐associated macrophages

Cancers are heterogeneous tissues with tumour‐infiltrating immune cells playing key roles in disease progression.^193^ Tumour‐associated macrophages (TAMs) are major components of the immune cells infiltrating solid tumours. They are a heterogeneous population often coexpressing pro‐ and anti‐inflammatory markers.^194^ While pro‐inflammatory functions could aid tumour cell elimination,^195^ immune‐suppressive TAMs are linked to worse prognosis due to their contribution to tumour growth and metastasis.^196^, ^197^ TAMs repolarization to produce inflammatory mediators is currently being evaluated in a number of clinical trials, examples of which are summarized in Table 1.^198^, ^199^, ^200^, ^201^, ^202^, ^203^, ^204^, ^205^, ^206^ Several strategies to target TAMs have been adopted, and we will describe common ones below.

**Table 1:** Selective examples of TAMs reprogramming compounds currently undergoing clinical trials updated

Compound	Target	Clinical phase	Clinicaltrials.gov identifier	Status	Results	Type of malignancy
Imiquimod, cyclophosphamide and radiotherapy	TLR7	Phase II	NCT01421017	Complete	No results available	Skin metastasis in breast cancer
Imiquimod together with Abraxane	TLR7	Phase II	NCT00821964	Complete	Pathologic clinical response in 71·4% of patients	Advanced breast cancer^200^
Imiquimod	TLR7	Phase II	NCT00031759	Complete	No impact on recurrence of cervical dysplasia	Cervical cancer^201^
Imiquimod	TLR7	Phase III	NCT00941252	Complete	Histologic regression in 73% of patients	Cervical intraepithelial neoplasia^202^
Imiquimod	TLR7	Phase III	NCT01861535	Active, not recruiting	‐	Vulvar intraepithelial neoplasia
Imiquimod	TLR7	Phase III	NCT02394132	Recruiting	‐	Complex lentigo maligna
Imiquimod	TLR7	Phase IV	NCT01161888	Complete	No results available	Lentigo malignant of the face
Resiquimod	TLR7/8	Phase I/II	NCT01676831	Complete	Significant improvements of treated lesions in 75% of patients, clearing of all treated lesions in 30%	Cutaneous T‐cell lymphoma^203^
852A	TLR7	Phase I	NCT00095160	Complete	No results available	Refractory solid organ tumours
852A	TLR7	Phase II	NCT00319748	Complete	Evidence of immune activation as evaluated by cytokine production	Breast, ovarian, endometrial and cervical cancers
852A	TLR7	Phase II	NCT00189332	Complete	No results available	Metastatic cutaneous melanoma
Imo‐2055	TLR9	Phase II	NCT00729053	Complete	Treatment‐emergent adverse events observed in > 90% of patients	Renal cell carcinoma
CD40 mAb CP‐870,893	CD40	Phase I	NCT02225002	Complete	No results available	Advanced solid tumours
CD40 mAb CP‐870,893 and gemcitabine	CD40	Phase I	NCT01456585	Complete	No results available	Pancreatic ductal adenocarcinoma^204^
CD40 mAb CP‐870,893 and chemotherapy	CD40	Phase I	NCT00711191	Complete	Partial response in 4/21 patients, stable diseases in 11/21 patients	Advanced cancer of the pancreas^205^
Vorinostat, gefitinib	HDAC	Phase I	NCT02151721	Unknown	‐	EGFR mutant lung cancer
IPI‐549 alone and with nivolumab	PI3Kγ	Phase I	NCT02637531	Recruiting	‐	Advanced solid tumours
IPI‐549 with Tecentriq and Abraxane/Avastin	PI3Kγ	Phase II	NCT03961698	Recruiting	‐	Breast cancer, renal cell carcinoma
IPI‐549	PI3Kγ	Phase II	NCT03795610	Recruiting	‐	Locally advanced HPV + and HPV‐ head and neck squamous cell carcinoma
IPI‐549 with nivolumab	PI3Kγ	Phase II	NCT03980041	Recruiting	‐	Advanced urothelial carcinoma
BLZ945 monotherapy or combination with PDR001	M‐CSFR (+/‐ PD‐1 blockade)	Phase I/II	NCT02829723	Recruiting	‐	Advanced solid tumours
PLX3397 + radiation therapy + temozolomide	M‐CSFR (+ cKit, Flt3)	Phase Ib/II	NCT01790503	Complete	Stable disease in 24/50 patients, complete response in 2/50, partial response in 5/50 patients	Glioblastoma
PLX3397 and sirolimus	M‐CSFR (+ cKit, Flt3)	Phase I/II	NCT02584647	Recruiting	‐	Unresectable sarcoma and malignant peripheral nerve sheath tumours
MCS110 with carboplatin and gemcitabine	M‐CSF	Phase II	NCT02435680	Complete	No results available	Advanced triple negative breast cancer with high TAMs
MCS110 with PDR001	M‐CSFR (+/‐ PD‐1 blockade)	Phase Ib/II	NCT02807844	Complete	Partial response in 1/48, stable disease in 9/48 patients	Advanced malignancies^206^
LY3022855	M‐CSFR	Phase I	NCT02265536	Complete	Stable disease in 5/22 MBC and 3/7 MCPRC patients	Metastatic breast cancer (MBC) Metastatic castration‐resistant prostate cancer (MCRPC)^207^

#### Receptor targeting

Just as in other tissues, the M‐CSFR axis is an attractive target in the tumour context: in many pre‐clinical models, the use of M‐CSFR inhibitors is associated with MФ depletion and tumour regression in a T‐cell‐dependent manner.^207^ However, blocking M‐CSFR signalling in murine proneural glioblastoma multiforme (PGM) and hepatocellular carcinoma caused tumour regression without TAM depletion, with persisting TAMs associated with functional alterations.^208^, ^209^ M‐CSFR signalling can be blocked by the use of antibodies targeting either M‐CSF or its receptor. While in some clinical trials antibody treatment achieved disease stabilization,^210^ other studies report TAM reductions without anti‐tumour activity.^211^

Another option is the use of small molecule inhibitors acting on the receptor tyrosine kinase domain (e.g. PLX3397, BLZ945). Those inhibitors also interfere with other receptors expressed in myeloid and tumour cells, which could enhance their efficacy. As it has been shown in a PGM model that tumours can acquire resistance to BLZ945‐mediated inhibition,^212^ the combination of M‐CSFR inhibitors with other immune‐ or chemotherapeutic agents could be beneficial and is being evaluated in clinical trials.

TLRs are involved in immune surveillance, and their agonists can drive pro‐inflammatory mediator release.^213^ Imiquimod (TLR7 agonist) is already approved for topical therapy in squamous and basal cell carcinoma, and clinical trials are ongoing for several other TLR agonists. Imiquimod’s mechanism of action likely affects not only MФ but also dendritic cells and neutrophils.^214^ *in vivo*, many TLR agonists have short half‐lives, and some free agonists have been associated with toxicity. Such issues could be prevented by encapsulation into nanovectors. While no such strategies are currently in clinical trials, murine models support the efficacy and safety of resiquimod‐loaded nanoparticles^61^ and ferumoxytol‐linked Poly(I:C).^215^

Another example of receptor targeting exploits CD40, a costimulatory molecule expressed on the surface of myeloid cells and B cells, with key roles in immune regulation.^216^ Ligation with an agonist CD40 mAb triggered T‐cell‐dependent anti‐tumour immunity in murine models of pancreatic ductal adenocarcinoma (PDAC) associated with increased MHCII, CD80 and CD86 expression on MФ and elevated serum levels of IL‐12 and TNF.^204^ Depletion of MФ in this context prevented tumour regression. In a clinical trial, CD40 mAb combined with chemotherapy showed a trend of increased overall survival, even though the sample size was too small (n = 21) for conclusive results.^204^ Several other clinical trials that investigate the effect of targeting CD40 in different types of tumours have already been summarized.^217^ While some of these therapeutics are associated with positive tumour outcomes, MФ‐specific effects of treatment are generally not investigated.

In a novel particle‐based strategy, Shields and colleagues injected MФ equipped with ‘backpacks’ of biodegradable polymers loaded with IFN‐γ into mice bearing 4T1 tumours; they showed that *ex vivo*‐polarized MФ carrying these backpacks were able to not only maintain a pro‐inflammatory phenotype in the tumour microenvironment, but also induce the same phenotype in resident TAMs, associated with reduced tumour growth, lung metastasis and improved survival.^64^ As backpacks could also be filled with cytokines promoting anti‐inflammatory polarization, such an approach could be useful in other disease contexts.

#### Intracellular pathway targeting

TAMs can also be targeted with compounds that interfere with internal signalling, including modifications of nucleic acids, cellular kinases or HDAC.

Vorinostat, a small molecule inhibitor of HDAC, is approved for cutaneous T‐cell lymphoma due to its growth‐inhibiting effect on tumour cells.^218^ Vorinostat also affects TAMs in murine models: encapsulating vorinostat and a chemotherapeutic drug within liposomes that target both non‐small‐cell lung cancer cells and CD206‐expressing MФ suppressed tumour growth via the upregulation of iNOS, CD86 and TNF while downregulating CD206, arginase and IL‐10 in MФ.^219^

Another internal target for influencing MФ signalling is PI3Kγ, a phosphoinositide 3‐kinases subunit mainly expressed by myeloid cells. In a murine model of PDAC, PI3Kγ blockade with the PI3Kγ/δ inhibitor TG100‐115 reduced MФ expression of Arginase1, TGF‐ß and IL‐10 and increased IL‐12 and IFN‐γ.^220^ This induced tumour suppression and prevented metastasis. MФ‐specific effects were replicated in a clinical trial of the selective PI3Kγ inhibitor IPI‐549 as monotherapy and in combination with chemotherapy, although the study is still ongoing and no results regarding survival and tumour regression are available yet.^221^

In mouse models of ovarian cancer, melanoma and glioblastoma, nanoparticle‐mediated delivery of *in vitro*‐transcribed mRNAs encoding IRF5 together with its activating kinase IKKβ increased pro‐inflammatory myeloid cells and caused tumour clearance in some animals.^222^ Similar results were found with nanoparticles delivering siRNA targeting growth factors including VEGF and placental growth factor, which reduced MФ CD206 expression and IL‐10 production while increasing IL‐12 and IFN‐y in tumour tissues.^223^ Recently, siRNA‐loaded nanoparticle mediated silencing of signal transducer and activator of transcription 3 and hypoxia‐inducible factor 1 α prevented TAM‐mediated angiogenesis and decreased tumour size.^224^ *In vitro*, repolarization has also been achieved with nanoparticle delivery of plasmid DNA encoding the IL‐12 gene^225^ and with overexpression of microRNA‐155 in triple negative breast cancer TAMs.^226^ Another approach delivers CRISPR‐Cas9 gene editing machinery into MФ: knock out of signal regulatory protein α, which engages CD47 on cancer cells and prevents phagocytosis, significantly increased MФ targeting of osteosarcoma cells *in vitro*.^227^

TAMs re‐education into pro‐inflammatory phenotypes can be beneficial to overcome tumour‐induced immune suppression. Reprogramming strategies can be combined with existing immunotherapies, chemotherapy and radiation, which has been shown to be successful in a variety of clinical trials. Importantly, TAMs targeting should be localized to cancerous areas to prevent systemic inflammation, and only affect pro‐tumoral MФ types. To achieve this, further investigation into TAMs heterogeneity and careful design and delivery of potential therapeutics are required.

## Discussion

The recent development of individual cell‐based technologies has led to an explosion of knowledge about MФ ontogeny and diversity. The identification of their subpopulations in homeostatic and disordered contexts is a constantly growing field that highlights a broad phenotypic repertoire consistent with the variety of stimuli they are exposed to. Their impact on disease development is still under intense investigation and seems to be subtype‐dependent. While some subsets are thought to directly contribute to disease progression, others have been found to be protective. However, most investigation is performed in animal models and the translation of this to human patients is often less clear.

Past approaches to MФ reprogramming did not appreciate MФ heterogeneity and plasticity, and how microenvironmental niche influences the phenotype. Early adoptive transfer studies had limited/no efficacy because of the impact of the *in vivo* microenvironment on MФ phenotype once transferred.^228^ Novel experimental avenues, however, have succeeded in maintaining the phenotype of adoptively transferred cells despite environmental factors.^64^ Other strategies have focused on direct *in vivo* modulation of MФ phenotypes, also giving promising results. While different approaches to MФ reprogramming have been described, each is associated with pitfalls and benefits influencing their utility for specific tissues. Where possible, future therapeutic approaches should consider tailoring of strategy towards a specific tissue microenvironment, as well as a specific disease‐associated subset of cells, to improve efficacy and minimize off‐target effects.

As MФ reprogramming is a recent approach to therapy, important questions still need to be answered: How stably can different subsets of MФ be re‐educated? Is long‐term reprogramming of long‐lived resident MФ safe? As MФ are in constant interaction with their environment and other immune cells, including other MФ subtypes, will modifying one particular subtype have secondary unintended impacts on the tissue? An alternative approach may be secondary targeting of MФ populations via manipulation of their environmental cues through alteration of the communication between tissue and MФ. This could be achieved by targeting the cells that support MФ growth and/or polarization, for example. Either way, it is clear that further growth in understanding of MФ phenotypic heterogeneity in a given microenvironmental/disease context is required to appreciate the potential of targeting the MФ in disease and capitalize on the advances that have begun to be made in this area.

## Competing interest statement

The authors declare no conflict of interest.

## Data Availability

Not applicable.
